# Shell color polymorphism in marine gastropods

**DOI:** 10.1111/eva.13416

**Published:** 2022-06-07

**Authors:** Juan Gefaell, Juan Galindo, Emilio Rolán‐Alvarez

**Affiliations:** ^1^ Departamento de Bioquímica Genética e Inmunología Centro de Investigación Mariña Universidade de Vigo Vigo Spain

**Keywords:** clines, crypsis, frequency‐dependent selection, genetic drift, natural selection, snails

## Abstract

Marine gastropods are characterized by an incredible variation in shell color. In this review, we aim to introduce researchers to previous studies of shell color polymorphism in this group of animals, trying to provide an overview of the topic and highlighting some potential avenues for future research. For this, we tackle the different aspects of shell color polymorphism in marine gastropods: its biochemical and genetic basis, its patterns of spatial and temporal distribution, as well as its potential evolutionary causes. In particular, we put special emphasis on the evolutionary studies that have been conducted so far to reveal the evolutionary mechanisms responsible for the maintenance of shell color polymorphism in this group of animals, as it constitutes the least addressed aspect in existing literature reviews. Several general conclusions can be drawn from our review: First, natural selection is commonly involved in the maintenance of gastropod color polymorphism; second, although the contribution of neutral forces (gene flow‐genetic drift equilibrium) to shell color polymorphism maintenance do not seem to be particularly important, it has rarely been studied systematically; third, a relationship between shell color polymorphism and mode of larval development (related to dispersal capability) may exist. As for future studies, we suggest that a combination of both classical laboratory crossing experiments and ‐*Omics* approaches may yield interesting results on the molecular basis of color polymorphism. We believe that understanding the various causes of shell color polymorphism in marine gastropods is of great importance not only to understand how biodiversity works, but also for protecting such biodiversity, as knowledge of its evolutionary causes may help implement conservation measures in those species or ecosystems that are threatened.

## 1 INTRODUCTION

Color polymorphisms are widespread in nature (White & Kemp, 2016). Several taxa are widely known for their rich and beautiful color patterns (Cuthill et al., 2017), sometimes showing a striking intrapopulation diversity. The term “polymorphism” is often used to refer discrete, genetic, and intrapopulation variation in a trait (Mayr, 1963), but here we will adhere to a more relaxed definition, as the intraspecific variation in a trait, whether it is geographically or temporally distributed, is more continuous than discrete, or if it is not directly dependent on genes. Color polymorphism and its maintenance have been a major topic of study in evolutionary biology since at least half a century (Clarke, 1962; Ford, 1965; Murray & Clarke, 1966). In the past decades, the number of studies of color polymorphism and its various aspects has grown exponentially (e.g., Bond, 2007; Gray & McKinnon, 2007; Jamie & Meier, 2020; McKinnon & Pierotti, 2010; McLean & Stuart‐Fox, 2014; Svensson, 2017; Takahashi & Noriyuki, 2019; Wellenreuther et al., 2014; White & Kemp, 2016).

The terrestrial snail *Cepaea nemoralis* stands out as a classic model organism for color polymorphism, showing highly diverse shell colors (reviewed in Cook, 2017). However, marine gastropods, despite being as diverse in their shell colors as *Cepaea*, and probably much more conspicuous, have been much less studied to date. In these organisms, shells serve different purposes, most of them related to fitness, such as protection from various abiotic factors or predators (e.g., Reid, 1996), and color could also be involved in some of those purposes. This, along with the fact that marine habitats are highly heterogeneous, cause the shells to be subjected to different selective pressures that, presumably, account for the high variability in shape, pattern, and color shown by these species, and that leads to shell polymorphisms.

In the present review, we summarize what is known about shell color polymorphism in marine gastropods, including its biochemistry and genetic basis, the patterns of diversity for this trait (intrapopulation, spatial, and temporal variation) and the evolutionary mechanisms responsible for the polymorphism. Our target audience are those researchers interested in shell color polymorphism in this group of animals, and especially those who are new to this research topic and who wish to be introduced to its study. There have recently been published several reviews regarding shell color (Williams, 2017) or shell color polymorphisms (Cook, 2017) in mollusks. However, these reviews hardly refer to color polymorphisms in marine gastropods as a whole. Since most of marine gastropods form a monophyletic clade, and the marine environment is very different from the terrestrial one, we believe that a review focused only on this group of organisms is necessary. Furthermore, both reviews, while valuable, do not analyze in depth the evolutionary mechanisms responsible for color polymorphism. For this reason, in this review, we place special emphasis on studies that have investigated these evolutionary mechanisms linked to color polymorphism on marine gastropods.

### 1.1 Search strategy

**FIGURE 1:**
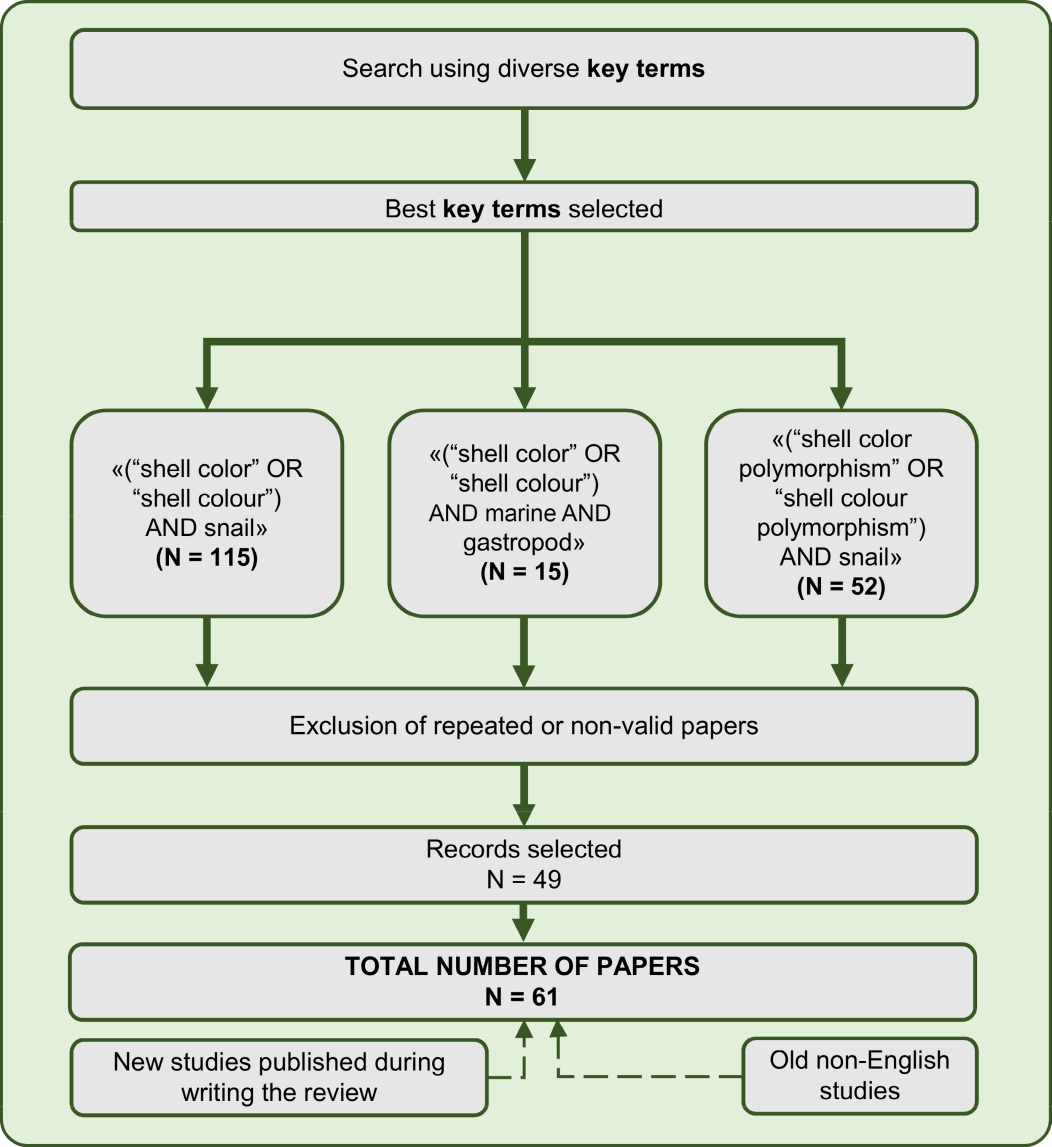
Flowchart of studies selected for the review

In January 2020, we searched for published papers on the topic “shell color polymorphism in marine gastropods” in the Web of Science (WOS). In a prospective search, we used various combinations of target terms, from which the three most effective combinations arose (see Figure 1). The first combination yielded 115 studies, the second 15 and the third 52. A total of 49 studies were kept after repeated or non‐valid papers (i.e., those that do not deal with shell color polymorphism in marine gastropods) were excluded. To this list, we added some references found in these studies, as well as a few old studies published in non‐English journals known by the authors and new studies published after January 2020, leading to a total of 61 studies. All of those studies were later classified on the basis of the intended structure of this review (Table 1).

## 2 THE COLOR OF SHELLS

### 2.1 A physical perspective

Colors stem from the pattern of reflection and absorption of electromagnetic radiation that a certain object shows. Reflected electromagnetic radiation can then be captured by the photoreceptors (usually cone cells) of the visual system of a given animal and transduced into nervous impulses that travel to the central nervous system, giving rise to color sensation. The different colors emerge from the different wavelengths (λ) of the reflected electromagnetic radiation. Every color‐sensible species has a range of visible wavelength spectrum (Cuthill, 2006). For humans, this visible spectrum, which encompasses all visible colors, ranges from wavelengths of 400 nm (violet) to 700 nm (red). Between those wavelengths are the rest of primary colors (blue, 450 nm; green, 525 nm; yellow, 575 nm; orange, 625 nm) and their derivatives. Many species, however, can go beyond the human visible spectrum: certain fish, birds, and many groups of arthropods can reach 320 nm, what is called the ultraviolet range, and some butterflies and fish can detect wavelengths of up to 760 nm (Gerl & Morris, 2008; Land & Nilsson, 2012). This information should be taken into account when designing experiments on the contribution of predation to the evolutionary maintenance of shell color polymorphism.

### 2.2 A biochemical perspective

At the molecular level, the color of a given organism is determined by the combination of the structural properties of their organs plus the effect of certain color biomolecules (“biological pigments”) contained in it. Those pigments have a particular range of absorbed and reflected electromagnetic energy, resulting in a specific visible color (Prum, 2006). In most marine gastropods, observable shell color relies heavily on the pigments present in its outer layer or periostracum.

Although knowledge on the biochemical basis of shell color polymorphism is still scarce, in the last decade several discoveries have been made in this regard. Current research has shown that the shells of marine gastropods can contain different classes of pigments, such as tetrapyrroles (porphyrins, bilins), carotenoids, and melanins. Porphyrins may be responsible for red, brown, and purple, while bilins have been involved in the determination of blue, green, brown, and red colors (Williams, 2017). For example, Williams et al. (2016) showed that uroporphyrins I and III are responsible for the pink‐red color of strawberry topshells (*Clanculus pharaonius*), as well as the pink‐red dots and lines of the early whorls and the yellow‐brown color of later whorls in the beautiful clanculus (*C*. *margaritarius*). Nevertheless, porphyrins have not been identified in other marine snails that show a similar coloration (e.g., the European painted top shell, *Calliostoma zizyphinum*), suggesting that the same color may be caused by different pigments in different species of marine gastropods (Saenko & Schilthuizen, 2021; Williams et al., 2016).

Carotenoids have not been directly characterized in marine gastropods, but some polyene compounds found in various species have been interpreted as indirect evidence of them (Williams, 2017). Furthermore, based on their chemical properties, Kozminsky and Lezin (2007) have proposed that the yellow coloration in the shell of the flat periwinkle (*Littorina obtusata*) could be caused by carotenoids. Given that marine gastropods cannot synthesize carotenoids, these pigments may be acquired from algae ingestion (Williams, 2017), which constitutes the diet of many marine snail species (including *L*. *obtusata*). Melanins may be responsible for dark shell colors, as well as for red, brown, and yellow pigmentation. In a classical study, Comfort (1951) suggested that the dark shell colors of the common periwinkle (*Littorina littorea*) may be caused by melanoproteins analogous to those of bird feathers. Finally, other biological pigments have been suggested as potential causes of shell color, such as guanine, associated by Kozminsky and Lezin (2007) to the white coloration of *L*. *obtusata* (see also Kozminsky, 2014).

### 2.3 A genetic perspective

Two hypotheses can account for the ontogenetic origin of shell color polymorphism in marine gastropods: (1) this trait may be caused by genetic differences in those genes that code for the biological pigments of the shell; (2) shell color is a plastic trait sensitive to different ecological factors, and therefore prone to showing polymorphic (i.e., polyphenic) variation. We will now consider the genetic hypothesis, leaving the phenotypic plasticity hypothesis for section 4.3.

Following Ruse’s (2006) distinction between direct and indirect evidence for evolution, we can separate between direct and indirect evidence for the genetic basis of shell color. We shall call *direct evidence* all scientific data regarding the metabolic pathway from genes to (color) phenotype, and *indirect evidence* the inferred information about the purported allelic (or quantitative) basis of shell color, as well as its pattern of transmission, based on laboratory crosses and the phenotypic analyses of parents and offspring. Ideally, the genetic basis of a given trait should be supported by both direct and indirect evidence.

Direct evidence for the genetic basis of shell color in marine gastropods is scarce: only two studies have been done so far on the gene‐to‐phenotype pathway for shell color (Koch et al., 2021; Williams et al., 2017). Williams et al. (2017) obtained direct evidence of the role of several enzyme‐coding genes in the production of pigments that are ultimately responsible for shell color in two species of the genus *Clanculus*. In particular, these genes coded for several enzymes of the pathway responsible for the synthesis of uroporphirine I and III (see previous section above).

In the second study, Koch et al. (2021) used a QTL (quantitative trait loci) approach with F_2_ families of the rough periwinkle (*Littorina saxatilis*), and they performed targeted sequencing to genotype the offspring for 25000 DNA fragments spread across the genome. The goal of the study was to map known adaptive traits between two divergent ecotypes of *L*. *saxatilis*, and although shell color was not shown yet to be adaptive, it varied between these ecotypes (beige vs black). Several QTLs for adaptive traits were associated with chromosomal inversions, similarly to previous studies (Westram et al., 2018), and QTLs for color were detected within two of these inversions. For example, a QTL for shell color was located in the same inversion as traits like weight, shell thickness, shell length, and aperture shape. Therefore, shell color might be evolving linked to traits under selection within a chromosomal inversion in these *L*. *saxatilis* populations, instead of being a direct target of selection. However, these results should be interpreted with caution, as QTLs represent large regions of DNA and no genes have been identified yet as responsible for this color polymorphism.

Indirect evidence for the genetic basis of color polymorphism in marine gastropods is more abundant. One of the first studies in this regard was carried out in the eastern oyster drill (*Uralpinx cinerea*; Cole, 1975). Results suggested that juvenile shell color is genetically inherited through a triallelic, single‐locus genetic system, in which a dominance hierarchy between the different alleles exists. Palmer (1984) found a similar trend for shell color polymorphism in the emarginate dogwinkle (*Nucella emarginata*). Similarly, crossing experiments carried out by Ekendahl and Johannesson (1997) on populations of *Littorina saxatilis* from Iceland point to a relatively straightforward genetic basis for band shell color, which in this case would be a one‐locus two‐allele system.

During the last decade, a series of noteworthy cross‐breeding studies on the various aspects of shell color polymorphism in the genus *Littorina* have been conducted by Kozminsky and collaborators (Kozminsky, 2011, 2014, 2016; Kozminsky & Lezin, 2007; Kozminsky et al., 2010). These researchers tested the different modes of inheritance of the various components of the shell color of *L*. *obtusata*: background color (Kozminsky, 2014), shell bands (Kozminsky, 2011, 2016; also *L*. *saxatilis* in Kozminsky, 2011) and the pattern of white spots (Kozminsky et al., 2010). Evidence suggested that simple Mendelian models successfully explained only some shell color components. For example, dark shell bands in *L*. *saxatilis*, and dark and white shell bands in *L*. *obtusata* were accounted for by a monogenetic biallelic system of inheritance (Kozminsky, 2011, 2016). However, background color in *L*. *obtusata* could not be explained by one or two Mendelian loci (Kozminsky, 2014). Given that background color in this species depends on several pigments, the author proposed that it can be caused by a gene system responsible for the separate inclusion of the different pigments in the shell.

Despite progress made in the last decade, to date, there are no marine gastropod species in which both direct and indirect inferences have allowed researchers to elucidate the proximate genetic mechanisms of color determination with as much detail as in other model organisms (see the peppered moth case reviewed in Cook & Saccheri, 2013).

### 2.4 A sensory ecology perspective

To understand shell color polymorphism, it is important to take into account the role of sensory ecology (Stevens, 2013), or the way in which marine gastropods and their predators process color vision. This knowledge is needed for the success of experimental designs intended at revealing the potential fitness advantages of different shell colors in polymorphic species.

Only a few studies have dealt with the visual abilities of marine gastropods (Hamilton et al., 1983; Seyer, 1992), and in these studies, no clues are offered on whether they have color vision. In any case, it is highly implausible that (at least most) marine gastropods can see colors. Cephalopods, the mollusks with a more advanced visual system (Nilsson, 2013) typically have one kind of photoreceptor, therefore not being able to see colors (with some exceptions; Land & Nilsson, 2012). If cephalopods, whose behavioral ecology depends much more on vision than that of marine gastropods (e.g., Hanlon & Messenger, 2018), cannot see in color, it is highly unlikely that gastropods can. However, an alternative hypothesis is that even color‐blind gastropods might be able to distinguish shell colors if these represent distinct levels of brightness/shading across the range from light to dark. Still, this hypothesis has not yet been tested in marine gastropods and would require further investigations.

Regarding the sensory ecology of predators, littorinid snails as well as other marine gastropods are usually preyed upon by intertidal crustaceans (such as crabs), shore birds (such as gulls and oystercatchers), pond fishes (such as blennies and gobies), and predator snails (such as the dog welk, *Nucella lapillus*; Pettitt, 1975). At least some species in these groups of predators (with the exception of the dog welk) have color vision, and evidence gathered in the past two decades suggests that some of them can even see beyond the human visible spectrum (see Marshall et al., 2003, 2019; Ödeen et al., 2010). This means predators may select their preys based on their visual properties, including those that go beyond the visible spectrum.

In sum, what we have seen throughout this section allows us to draw three conclusions: (1) the biochemical basis of shell color polymorphism depends on a limited number of pigments that vary between species; (2) simple Mendelian models can account for many shell color traits but molecular and physiological mechanisms are unknown; and (3) while marine gastropods cannot see color, most of their predators can.

## 3 PATTERNS OF DIVERSITY

### 3.1 Intrapopulation variation

**FIGURE 2:**
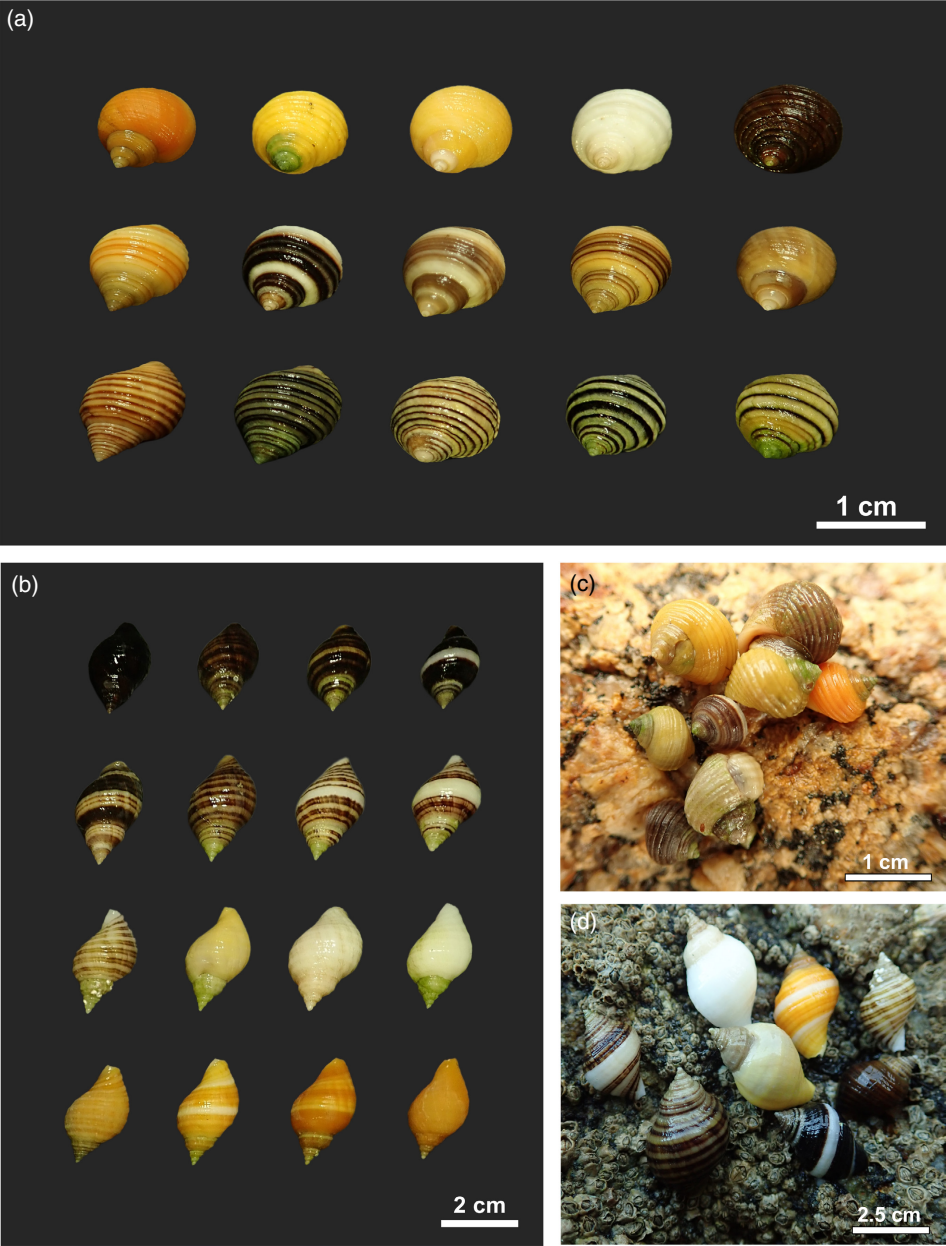
(a) Color polymorphism shown by *L*. *saxatilis*. (b) Color polymorphism shown by *Nucella lapillus*. (c) Some color morphs of *L*. *saxatilis* in their natural environment. (d) Some color morphs of *Nucella lapillus* in a natural setting

Shell color variation within populations is a common phenomenon in marine gastropods (see Figure 2). Perhaps, one of the most studied groups in this respect is the genus *Littorina*: within this genus at least five of 18 species show a clear and conspicuous intrapopulation shell color polymorphism, with the rest of the species showing only very subtle intrapopulation color variation (reviewed in Reid, 1996; Rolán‐Alvarez et al., 2015a). *L*. *saxatilis* shows shell color variation within several populations of the Galician coasts (NW Spain), including orange, white and banded color morphs (Fischer‐Piette & Gaillard, 1961; Fischer‐Piette et al., 1961; Gefaell et al., 2021; Sacchi, 1979; Torelli, 1983). Northern populations of *L*. *saxatilis* in the British Isles, Sweden, Iceland, and Russia also show great intrapopulation polymorphism (e.g., Ekendahl & Johannesson, 1997; Raffaelli, 1979).

Regarding *L*. *obtusata* and *L*. *fabalis*, most studies have detected the existence of four different color morphs (brown, olive, yellow, and orange) that usually coexist in the same populations (Estévez et al., 2020; Gefaell et al., 2021; Reid, 1996; Rolán‐Alvarez et al., 2012, 2015b). Hughes and Jones (1985) recognized three color morphs (yellow, red, and brown) coexisting in populations of *Littorina scabra* (now *Littoraria scabra*) from Queensland (Australia). In addition, patterns of intrapopulation shell color polymorphism have been described in species from genera other than *Littorina* (for instance, *Hastula cinerea*; Molina et al., 2013); colors beyond the visible spectrum have also been described, such as the near ultraviolet, which may be detected by some predators of snails and therefore subject to selection (Williams, 2017; see previous section). Based on current data, it is highly likely that most marine gastropods show at least a subtle degree of intrapopulation shell color variation. However, the prevalence of conspicuous intrapopulation shell color polymorphism in marine gastropods, as well as its potential correlation with developmental characteristics, genetic variation, and environmental variables, has not been yet systematically analyzed (an exception to this is the genus *Littorina*, Reid, 1996).

### 3.2 Spatial variation

Shell color variation occurs not only within populations, but also between populations, showing spatial patterns. When this type of color variation is gradual, it is called *cline* (*sensu* Mayr, 1963). Clinal variation in shell color between populations has been observed in *L*. *saxatilis*, where yellow and fawn individuals are abundant in sheltered localities of estuaries, and gradually decrease in frequency as we approach the exposed shores, where they are almost completely replaced by a banded phenotype (Reid, 1996; Sacchi, 1979; Sacchi & Malcevschi, 1983 and references therein). A similar kind of spatial variation has been found in *Tegula xanthostigma*, in which light brown shells were found to be associated with sheltered environments, and black shells with exposed shores (Yamazaki et al., 2019).

In other occasions, clines may show gradual variation in the number of color morphs present at a given location. Several *Littorina* species (*L*. *obtusata*, *L*. *fabalis*, and *L*. *compressa*) have been reported to show higher numbers of color morphs in the southern parts of their ranges than in the northern parts (Reid, 1996). In a similar vein, Magnúsdóttir et al. (2018) found that in the north‐Atlantic common whelk (*Buccinum undatum*), populations in the innermost parts of a bay located in western Iceland showed low color diversity than in the outer parts. This suggests that natural selection and other evolutionary mechanisms somehow promote the evolution of a higher degree of color variation (seen in the number of color morphs available) in certain locations compared to others, but the exact reasons for this pattern are not clear.

### 3.3 Temporal variation

The most important temporal variation in color involves changes in the frequency of the different colors in the population over time. For example, in *L*. *fabalis* seasonal variation in shell color (yellow, brown) was studied in Swedish populations, showing that color frequencies remained similar between spring and autumn (Ekendahl, 1994, 1995). However, these studies revealed several microhabitat changes that could potentially affect survival. For instance, Ekendahl (1995) found that in autumn, yellow adult morphs were more frequent in the laminae of the seaweed *Fucus vesiculosus*, while brown adults were more frequent in stipes. On the contrary, in spring no differences were observed between adult morphs. These results suggest a seasonal pattern of habitat choice that could be linked to different selective pressures affecting the color morphs in the different seasons.

Besides seasonal variation, other studies have looked for monthly or annual variations in color frequencies (Estévez et al., 2020, 2021; Tursch et al., 1995). This is the case of *L*. *fabalis* (Estévez et al., 2020, 2021), which did show a reduced change in frequencies over a hypothetical equilibrium frequency in the course of 20 years. However, larger time spans have not yet been analyzed.

### 3.4 Variation across taxa

It is not easy to find patterns in shell color polymorphism across gastropod species because the number of known polymorphic species is relatively low (27 species in Table 1). However, at least a few preliminary suggestions can be made. For example, it has been suggested that the pattern of larvae dispersal (direct *versus* pelagic) could influence the level of population genetic (and, therefore, color) variability (Bohonak, 1999). In species with direct development, eggs are laid in the immediate surroundings of the mother (or carried by the mother until they are mature, as in ovoviviparous species such as *L*. *saxatilis*); pelagic species are those that go through a planktonic larval phase until metamorphosis, when they return to the shore (Hickman et al., 2007).

According to Table 1, the number of polymorphic species for shell color that are direct developers is higher than those with pelagic larvae (59.3% vs. 40.7%). This pattern seems to change if we broaden our focus to marine gastropods in general, whether polymorphic or not; in this group of animals, 45% undergo direct development, while about 55% of them show a pelagic larvae stage (Jablonski & Lutz, 1983). This suggests that direct development could somehow promote shell color polymorphism. This is further supported by the averaged maximum number of shell colors observed in direct developers (mean ±SD = 7.38 ± 7.41, N = 13) *versus* those with pelagic larvae (3.37 ± 1.92, N = 8). In addition, it has been suggested that phenotypic plasticity is more prevalent in organisms with high dispersal ability (Hollander, 2008). However, plasticity in shell color polymorphism is similarly distributed in direct (4 out of 16) and pelagic species (3 of 16) of marine gastropods.

## 4 EVOLUTIONARY MECHANISMS

After reviewing the “How” and “What” of color polymorphism, that is, its physicochemical nature, genetic basis, and patterns of diversity, it is now time to review the “Why” of color polymorphism, or, in other words, the evolutionary mechanisms responsible for their evolution and maintenance within populations (Mayr, 1998).

**FIGURE 3:**
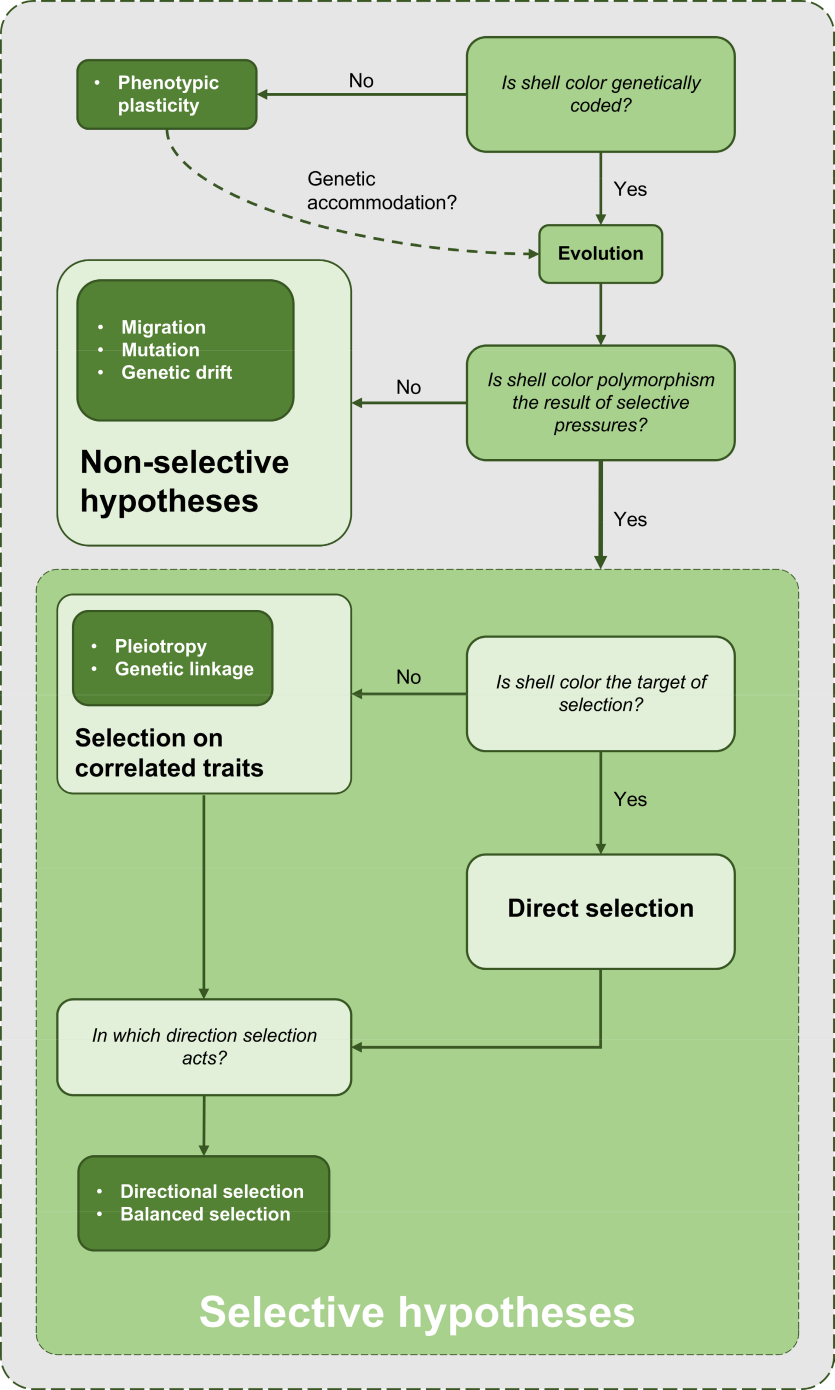
A summary of all proposed hypotheses to account for shell color polymorphism portrayed in an algorithm‐like fashion. See text above for a detailed explanation

Several different hypotheses have been proposed to account for color polymorphism (see White & Kemp, 2016). We have arranged these hypotheses following an algorithm‐like scheme (see Figure 3). Once we have put aside the special case of phenotypic plasticity, the main distinction can be drawn between non‐selective and selective hypotheses. In turn, selective hypotheses can be further subdivided into direct selection and selection on correlated traits. In addition, a distinction can also be drawn between directional and balancing selection.

### 4.1 Non‐selective hypotheses

Three evolutionary mechanisms promote the evolution of populations besides selection: mutation, migration, and genetic drift (Freeman et al., 2017). Mutations introduce new (color) variations in a given population. Although it is reasonable to assume that several color polymorphisms are caused by mutations in genes involved in shell coloration, no particular mutation for shell color has yet been detected at the molecular level in marine gastropods.

Migration refers to the reproductive consequences of the transfer of one or many individuals from one population to another. From an evolutionary point of view, migration is important insofar as it contributes to gene flow, causing the homogenization of populations, thereby making it harder for specific adaptations to evolve (Freeman et al., 2017; Herron & Freeman, 2014). Evidence suggests that direct developers show a greater tendency toward evolving local adaptations than their planktonic counterparts (Behrens Yamada, 1989).

Many stochastic changes in the genetic makeup of populations are due to genetic drift (see Freeman et al., 2017; Futuyma, 2009; Herron & Freeman, 2014). This mechanism explains the fixation of alleles that do not bear a reproductive advantage as a result of sampling error during mating. Among the non‐selective mechanisms, genetic drift has classically stood out as a plausible explanation for the occurrence of phenotypic polymorphisms; for instance, Darwin himself noted in *On the Origin of Species* (1859/2006) that: “Variations neither useful nor injurious would not be affected by natural selection, and would be left a fluctuating element, as perhaps we see in the species called polymorphic.” (1859/2006: 63–64).

The equilibrium between genetic drift and migration should be the null hypothesis when testing the role of other evolutionary mechanisms, particularly selection. Genetic drift assumes that the different color variations (whether intrapopulation or clinal) do not bear any fitness advantage, so they fluctuate in a random—but quantifiable—manner across locations and generations. In intrapopulation polymorphism, genetic drift and migration equilibrium can be rejected if the observed intergeneration allele variance is higher or lower than the one predicted based exclusively on effective population size (*N_e_*; assuming absence of effective migration; see for example Estévez et al., 2020, 2021). In the case of clinal polymorphism, genetic drift and migration are expected to lead to unique and non‐repeatable patterns of color variation at different locations (although the magnitude of variation across subdivided subpopulations can be to some extend predicted; see for example Johannesson & Butlin, 2017). This means that, if a given clinal polymorphism is observed in a similar ecological context in several (and at least partially isolated) locations, then the null hypothesis can be rejected (or at least considered highly unlikely).

Although most research tacitly assumes the equilibrium of genetic drift and migration as null hypothesis, very few attempts have followed this framework in the study of shell color polymorphism. Besides the examples cited above (Estévez et al., 2020, 2021; Johannesson & Butlin, 2017), another exception is Cook (1992), who indirectly tested genetic drift in the mangrove snail *Littoraria pallescens* by estimating the effective population size (*N_e_*) either based on molecular markers or color variation. As the level of diversity for both genetic traits could be explained based on correspondingly estimated *N_e_*, he concluded that color variation may be a trait as neutral as molecular variation, although he admitted some local exceptions putatively caused by selection (Cook, 1992). In any case, most previous studies (e.g., Cook, 1992; Estévez et al., 2020, 2021; Johannesson & Butlin, 2017) suggest that genetic drift and migration may not be a particularly important evolutionary mechanism for the explanation of shell color polymorphism in at least littorinid snails, but given that such mechanism is always working in nature (because *N_e_* ≠ ∞), it should be properly tested in each putative case before being formally ruled out.

### 4.2 Selective hypotheses

Natural selection is considered the most important evolutionary mechanism by the majority of evolutionary biologists, and it is frequently invoked as an explanation to account for all complex traits that resemble an adaptation. Although selective explanations should be formulated with caution, given the widespread tendency to incur what Gould and Lewontin (1979) called the “Panglossian paradigm,” its explanatory potential is undeniable (Mayr, 1983). Regarding shell color polymorphism in marine gastropods, an overwhelming majority of studies have assumed that natural selection is ultimately responsible for its evolution.

Natural selection can take many forms. A first distinction is based on whether a given trait (in our case, shell color) is the real target of selection. If this is so, then we can speak of direct selection; if, on the contrary, selection does not act directly on shell color, but on any other trait correlated with shell color, then we can speak of selection on correlated traits (Figure 3). Whether selection acts directly on shell color or on any other correlated trait, a further distinction can be drawn between directional and balancing selection. In directional selection, the direction of selection is constant in a given habitat during a significant number of generations, favoring those phenotypes at an initially lower frequency in the population; in balancing selection, selection acts in such a way that multiple variants in a population are maintained at larger frequencies than those expected by genetic drift. This can be ultimately achieved either by heterozygote advantage or frequency‐dependent selection.

#### 4.2.1 Directional selection

Directional selection can promote shell color polymorphism if there exists habitat heterogeneity in which the different color morphs bear different fitnesses. When selection results from visual predators, then crypsis could evolve and shell color will tend to match its background (Bond, 2007; Endler, 1978); if this background is heterogeneous, then color polymorphism can be expected, maybe in conjunction with a behavioral mechanism of habitat choice (e.g., Edelaar et al., 2008). In other words, the main prediction of directional selection is that color polymorphism must reflect the chromatic heterogeneity of a given habitat.

In the past, many studies in marine gastropods have evaluated directional selection using frequentist‐observational approaches that compare shell colors with their backgrounds (Byers, 1990; Ekendahl & Johannesson, 1997; Heller, 1975, 1979; Hoagland, 1977; Reimchen, 1979, 1989). For instance, Heller (1975) studied the frequencies of different colors in *Littorina saxatilis* from Wales. Among other things, he observed that reddish individuals were more prevalent in those localities where red sandstone was more abundant, suggesting that visual predation by rock pipits and crabs may be the main selective pressures there. Also in Welsh populations, Byers (1990) found that grey *L*. *saxatilis* individuals were more frequent in grey rocks, while brown individuals were more frequent in red rocks. One further example is that of *Littorina fabalis´* juveniles, which are cryptic with *Spirorbis* tubeworms, both found in the fronds of brown macroalgae, in order to avoid predation by blennies (Reimchen, 1989). These correlations between shell and background colors constitute powerful suggestions for crypsis, though not a definite corroboration, so manipulative experiments need to be carried out. For example, Ekendahl (1998) conducted tethering experiments in Swedish populations of *L*. *saxatilis* to test if crypsis was influenced by predation by the crab *Carcinus maenas*. For this, dark and white snails were glued to black and greyish stones, and then the number of preyed snails was counted. Predation results showed no differences in the number of preyed snails of both colors on each type of stones, suggesting that crabs hardly influence the color frequencies of these populations of *L*. *saxatilis*.

In another study, Hughes and Mather (1986) carried out mark‐recapture and caging experiments on the various color morphs of the mangrove snail *Littorina scabra* (now *Littoraria scabra*), that lives in trees of the species *Avicennia marina*, which have a mosaic of colors in their trunks and leaves, ranging from brown to yellow. This species of mangrove snail presents three background colors (brown, yellow and red, in decreasing order) and a striped pattern that can superimpose on background color. The mark‐recapture and caging experiments suggested that yellow individuals showed less predation and a higher survival. For instance, when the snails were placed at the cages, where predation by toadfishes (*Spheroides hamiltoni*) is restricted, yellow individuals decreased in frequency, whereas on control trees, with no cages preventing predation, the frequency of this morph increased. This suggests that yellow individuals are in one way or another protected against predators. Although Hughes and Mather did not rule out the existence of frequency‐dependent selection, they suggest that selection for crypsis may play a role in explaining the frequencies of observed color morphs in this species of mangrove snail.

So far, we have assumed that selection for crypsis is caused by predators; however, a series of studies point to the possibility that parasitoid insects may be responsible for the crypticity of certain color morphs in *Littoraria filosa* mangrove snails (McKillup & McKillup, 2002; McKillup et al., 2000). *Sarcophaga megafilosia* and *S*. *meiofilosia* deposit larvae near the snails, so that they can infect the snails and feed on them (eventually killing them). Results by McKillup and colleagues showed that *S*. *megafilosia* infects more those snails that do not match their background color, while *S*. *meiofilosa* does not differentiate between colors. These results suggest that at least one of these parasitoid species (*S*. *megafilosia*) could be maintaining color polymorphism in *L*. *filosa* due to habitat color heterogeneity. Further studies have supported this interpretation (McKillup & McKillup, 2008).

In addition to crypsis, other selective pressure that can promote directional selection on shell color is thermal stress. Shell color is presumed to contribute to thermoregulation, with light colors preventing overheating, while darker shells attracting heat. Therefore, if there is a thermal cline at a given region, then a color polymorphism based on thermal tolerance can be expected: colors less prone to overheating should be more prevalent in the warmer regions. Several studies have observationally and experimentally explored this possibility in various intertidal gastropods (Cook, 1986; Etter, 1988; Harris & Jones, 1995; Miller & Denny, 2011; Phifer‐Rixey et al., 2008). For example, in the Japanese mud snail (*Batillaria attramentaria*), darker morphs are predominant in colder regions, where almost no light‐colored individuals exist, suggesting strong selection against them (Miura et al., 2007). In warmer regions, lighter individuals are more frequent, but dark ones are still found, suggesting weaker selection against dark individuals in these regions. Similarly, Phifer‐Rixey et al. (2008) found that in *L*. *obtusata* from the Gulf of Maine the darkest individuals are found in colder regions, while the lighter ones are more frequent in warmer regions.

Experimental evidence also provides support for the thermal stress hypothesis. In a series of lab and field experiments conducted with dogwhelks (*Nucella lapillus*) from the northern coast of Massachusetts, Etter (1988) found that the brown morphs heated up faster and were less resistant to thermal stress than the white morphs in those locations with higher temperatures (the more protected ones).

The above results suggest that selection related to thermal stress may be a potential cause of the evolution of shell color polymorphism, also indicating a possible role for this trait in resilience to climate change. However, these results should be interpreted with caution as, for example, in the past decade Miller and Denny (2011) have shown that the snail's behavior makes a greater contribution to thermoregulation than shell color in the case of five littorinid species from California, based on field experiments and heat‐budget models. That is, to prevent overheating, behavioral mechanisms tend to be much more efficient than shell colors.

#### 4.2.2 Balancing selection

Balancing selection can promote polymorphisms through heterozygote advantage and frequency‐dependent selection. Heterozygote advantage occurs in those occasions in which heterozygote individuals show higher fitness than any of the two homozygotes (Futuyma, 2009). Frequency‐dependent selection occurs when the frequency of a given trait in the population affects the fitness of its bearer organism. The most striking case of frequency‐dependent selection is *negative* frequency‐dependent selection (hereinafter NFDS), in which a trait or phenotype increases its fitness as its frequency decreases (Ayala & Campbell, 1974; Lewontin, 1974). Since the classic studies on shell color polymorphism in *Cepaea* (Clarke, 1962), NFDS has been invoked as a putative explanation for color polymorphisms in a wide variety of organisms (Gigord et al., 2001; Olendorf et al., 2006; Estévez et al., 2021; but see Ibarra & Reader, 2013). The most common case of NFDS is *apostatic selection*, where the rarer phenotypes of a prey species show greater fitness than the more frequent phenotype because certain predators look for prey by forming a “search image” of its most common phenotype, thus ignoring those phenotypes at a lower frequency (Ayala & Campbell, 1974; Clarke, 1962).

In the case of shell color polymorphism, apostatic selection predicts that the color morph or morphs at lower frequencies are less detected by predators because they do not match the predator's search image. Thus, though present at low frequencies, these rarer morphs are protected from extinction by NFDS. Several potential cases of apostatic selection have been described to account for shell color polymorphism (Atkinson & Warwick, 1983; Johannesson & Butlin, 2017; McKillup & McKillup, 2008; Reid, 1987). For example, correlations between the frequencies of different color morphs, as well as between the levels of phenotypic diversity and the presence/absence of color morphs between *Littorina saxatilis* and *L*. *arcana* across several British populations were proposed as an indication of NFDS (Atkinson & Warwick, 1983). In the case of *Littoraria filosa*, and in addition to selection for crypsis by parasitoid insects, as we previously mentioned, evidence was also obtained for apostatic selection on color driven by shell‐crushing predators (most likely crabs) (McKillup & McKillup, 2008), suggesting that in some occasions different modes of natural selection may be operating simultaneously in opposing directions in a given species.

NFDS can also be driven by sexual selection, and among these scenarios, we find sexual conflict (Gosden & Svensson, 2007; Svensson et al., 2005), mate competition (Rios‐Cardenas et al., 2018; Sinervo & Lively, 1996), and mate choice (Takashi & Hori, 2008). However, in organisms with a limited behavioral repertoire, such as most marine gastropods, only mate choice has been linked to NFDS for shell color (Estévez et al., 2020; Gefaell et al., 2021; Rolán‐Alvarez & Caballero, 2000; Rolán‐Alvarez & Ekendahl, 1996; Rolán‐Alvarez et al., 2012, 2015b).

In the genus *Littorina*, negative assortative mating has been used as an indirect proxy for mate‐choice‐driven NFDS (see Gefaell et al., 2021 and references therein). Rolán‐Alvarez et al. (2012, 2015b) collected mated and unmated individuals of *L*. *fabalis* from different microareas to determine whether a process of sexual selection or assortative mating could be playing a role in the evolutionary maintenance of shell color polymorphism. These researchers found a high level of negative assortative mating affecting the mating behavior of this species; a result that was consistent with that found 21 years earlier in the same population (Rolán‐Alvarez & Ekendahl, 1996).

In a more recent study including seven consecutive years of analysis of a population of *L*. *fabalis*, Estévez et al. (2020) found both negative assortative mating and NFDS (via sexual selection) on shell color along the period studied, thus contributing to the maintenance of a polymorphism in this trait. Estévez and colleagues also used multi‐model inference to determine whether mate choice, mate competition or both could explain the observed distribution of shell colors observed for mating pairs, showing that mate choice was always included in all potential explanatory models (Estévez et al., 2020). Recently, similar patterns of negative assortative mating have been found in other populations of *L*. *fabalis*, as well as in *L*. *obtusata* and *L*. *saxatilis*, suggesting that male mate choice may be an ancestral behavioral trait driving NFDS on shell color, and thereby promoting polymorphism in at least certain populations of these species (Gefaell et al., 2021). However, since the relationship between negative assortative mating, mate choice and NFDS is not straightforward and direct, experimental confirmations of this link must be made before the mate choice‐driven NFDS hypothesis is considered definitively corroborated.

#### 4.2.3 Selection on correlated traits

The former selective hypotheses have assumed that natural selection acts directly on shell color; however, there could be cases in which the real target of selection may not be shell color itself, but any other correlated trait that may be the one which really bears a fitness advantage for the organism. Notice that in this case the mode of selection also occurs by either directional or balancing selection, as in the previous cases. We shall call this group of hypotheses *correlated traits’ selection hypotheses* (CTSH).

Two genetic mechanisms have been proposed as explanations for CTSH: linkage disequilibrium and pleiotropy (see McKinnon & Pierotti, 2010). In linkage disequilibrium, two given alleles are non‐randomly associated; on the other hand, in pleiotropy one gene is directly or indirectly responsible for the expression of two phenotypically independent traits. Irrespectively of the genetic mechanisms involved, CTSH predicts a correlation between shell color and some other phenotypic trait, which has to demonstrate some fitness advantage for the carrying organism. If such correlation and fitness advantage are found, then we will be ready to study its genetic and molecular basis.

A few putative examples of CTSH involving shell color polymorphism have been proposed for marine gastropods. It has been suggested that shell color polymorphism may be associated with parasite resistance (Raffaelli, 1979), shell strength (Cook & Kenyon, 1993), and haline stress (Sokolova & Berger, 2000; see Sergievsky, 1992 for an early review of Russian studies). For instance, Cook and Kenyon (1993) tested the shell strength of different color morphs in *Littoraria pallescens*, and they found differences in shell resistance between these colors. More specifically, dark shells of two of three analyzed locations were found to be heavier and more resistant. This greater resistance was dependent on the structural properties of this morph's shell and not on the color itself. Cook and Kenyon (1993) associated this difference in shell thickness with crab predation, which it is probably more prevalent in bark and rock surfaces, where the dark morphs live.

Another putative example of CTSH is found in *L*. *saxatilis*, where a color gradient has been observed in the White Sea, with brown tessellated unbanded (BTU) shells being more frequent in the head of the estuaries, where there is low salinity. Laboratory experiments revealed that BTU morphs show higher survival rates under extremely low salinity conditions and under a combination of low salinity and low temperatures compared to typical morphs from marine environments (Sokolova & Berger, 2000). This points to the possibility that these physiological differences associated with the BTU morph may be the result of adaptation to haline stress. However, an important issue with this hypothesis is having color and population as confounded factors in the analysis, as both color morphs may differ in several other traits simultaneously. Therefore, future studies comparing colors from the same population are needed before the final confirmation of this trend.

A third presumed case of CTSH involves mate choice and NFDS. Estévez et al. (2020), as well as Gefaell et al. (2021), have suggested the possibility that in the abovementioned cases of negative assortative mating and mate choice for shell color in several species of the genus *Littorina*, the real target of (sexual) selection may not be the color itself, but another genetically correlated trait, tightly linked to it. An argument in favor of this hypothesis, in addition to the observed mating patterns in different *Littorina* populations (see Gefaell et al., 2021), is that it is highly unlikely that marine gastropods can see colors, a prerequisite for mate choice based on shell color. This has led to the hypothesis that littorinids can somehow recognize the different shell colors without any visual cue, or alternatively each of these colors may be associated with some other trait crucial to biological fitness. There is already evidence that at least certain littorinid species can detect with olfactory cues their conspecifics of the opposite sex (Seuront & Spilmont, 2015; Wyeth, 2019). However, it cannot be ruled out that color‐blind snails could distinguish between colors based on the different brightness/shading effect produced by each color, so further studies are needed to test this possibility.

In cases such as the latter, the evolutionary mechanism at work may be heterozygote advantage. Although heterozygote advantage should be considered to affect to a minority of all loci in any species (Hedrick, 2012), this does not preclude that it may have a role in maintaining certain genes in some populations. A particular way in which this mechanism could have a role in maintaining shell color polymorphism could be by means of an association between shell color genes and chromosomal inversions. Chromosomal inversions can become overdominant under several scenarios (Kirkpatrick, 2010). Furthermore, chromosomal inversions have been recently detected in *L*. *saxatilis* associated with adaptative traits (Faria et al., 2019; Westram et al., 2018) and particularly to shell color (Koch et al., 2021). Therefore, heterozygote advantage should be further studied in relation to these findings.

### 4.3 Phenotypic plasticity

Phenotypic plasticity is usually defined as the process by which one individual genotype is able to produce different phenotypes under different environmental conditions (see Fusco & Minelli, 2010). It has been suggested that, at least in some occasions, phenotypic plasticity can precede evolutionary change; that is, variations arising via phenotypic plasticity could later be fixed at the genetic level, thus being inherited and subject to evolutionary forces (Ehrenreich & Pfennig, 2016; Gilbert & Epel, 2009; West‐Eberhard, 2003). This process, usually called genetic assimilation, has not yet been described in marine gastropods. However, some putative examples have been suggested in other taxons (e.g., Aubret & Shine, 2009; Badyaev et al., 2017), so the possibility of genetic assimilation in marine gastropods should not be ruled out. A first step toward investigating genetic assimilation is to corroborate the existence of phenotypic plasticity. In the present case, phenotypic plasticity predicts that shell color in a given population of snails is dependent on a given environmental parameter, thus changing if that parameter changes.

Most of the known cases of color polymorphism in marine gastropods caused by phenotypic plasticity are induced by changes in diet (Lindberg & Pearse, 1990; Liu et al., 2009; Manríquez et al., 2009; Palmer, 1984; Sorensen, 1984; Sorensen & Lindberg, 1991). This is the case of the black limpet (*Lottia asmi*) and the shield limpet (*L*. *pelta*), that show a large diversity of shell colors and patterns, securing cryptic coloration at the different types of substrates in which they live. It has been found that these colors depend on the type of diet available on each substratum, and that a change in the substrate (and therefore diet) triggers new shell growth that makes the limpet more cryptic in their new environment (Lindberg & Pearse, 1990; Sorensen, 1984; Sorensen & Lindberg, 1991). Another example is the so‐called Chilean abalone, *Concholepas concholepas* (a muricid gastropod), which has a shell color that matches the background color of their habitat, made up of their most frequent prey: black individuals feed on mussels and light colored on barnacles (Manríquez et al., 2009). Juvenile individuals reared in a specific habitat in the laboratory developed a shell color that matched this habitat. Moreover, when the individuals were moved to a different habitat, they showed a color change that corresponded to that of the new habitat. In sum, shell color in marine gastropods is sometimes dependent on phenotypic plasticity. Plastic shell colors may contribute to camouflage in a given habitat, so phenotypic plasticity could potentially interact with selection by crypsis. It remains open whether these initially plastic traits can eventually be genetically assimilated (Table 1).

**TABLE 1:** Summary of studies that have investigated shell color polymorphism in marine gastropods

Species	Nº of morphs	Color trait^a^	Genetic candidate^b^	Pleiotropic effects	Biodiversity^c^	Evolutionary factor^d^	Ecological mechanism^e^	Methods^f^	Larval dispersion mode^g^	References
*Littorina saxatilis*	2–13	BC, BA	MGB, TGB, Linkage Group 6 and 17	Thickness, Salinity toleration, Weight, Aperture shape	SPA, SNL, IP	DS, NFDS, PS, BS	SC, APS, SHS	F, ME, CE, MP, ‐*Omics*	D	Atkinson and Warwick (1983), Byers (1990), Ekendahl (1998), Ekendahl and Johannesson (1997), Estévez et al. (2021), Fischer‐Piette and Gaillard (1961), Fischer‐Piette et al. (1961), Gefaell et al. (2021), Heller (1975), Hughes and Jones (1985), Johanneson and Ekendahl (2002), Johannesson and Butlin (2016), Koch et al. (2021), Kozminsky (2011), Raffaelli (1979), Sacchi (1979), Sacchi and Malcevschi (1983), Sokolova and Berger (2000), Torelli (1983)
*Littorina fabalis*	2–6	BC, BA	MG, DG	Mucus	SPA, IP, SNL, ONT	DS, NFDS, PS, HS, SS	DM, APS, SC	F, ME, MP, CS, RS	D	Ekendahl (1994, 1995), Estévez et al. (2020, 2021), Gefaell et al. (2021), Reimchen (1979, 1989), Rolán‐Alvarez and Ekendahl (1996), Rolán‐Alvarez et al. (2012), Rolán‐Alvarez, Carvajal‐Rodríguez, et al. (2015b)
*Littorina obtusata*	2–4	BC, BA	MGB, DG	Several behav. and morphol. traits (unspecified)	IP, SPAGR	DS	STS	ME, CE, MOC, ‐*Omics*	D	Kozminsky (2011), Kozminsky (2014), Kozminsky (2016), Kozminsky et al. (2010), Phifer‐Rixey et al. (2008)
*Littorina compressa, Littorina arcana*	5, 10–13	BC/BA	ns	ns	SPA, IP	DS, NFDS	SC, APS	F	D	Atkinson and Warwick (1983), Byers (1990), Heller (1975)
*Littorina (Littoraria) scabra*, *Echinolittorina natalensis*	3–4	BC	ns	ns	IP	DS/NFDS	SC/APS, ev. against STS	ME, MP	P	Hughes and Jones (1985), Hughes and Mather (1986), Miller and Denny (2011)
*Littoraria filosa*	3	BC	ns	ns	SPA/IP	DS/NFDS	SC/APS	ME, F	P	McKillup et al. (2000), McKillup and McKillup (2002, 2008), Parsonage and Hughes (2002), Reid (1987)
*Littoraria pallescens*	2	BC	ns	Thickness	IP	DS, GD, PS	ns	F, ME, MP	P	Cook (1986, 1992), Cook and Kenyon (1993)
*Littoraria luteola*	3	BC	ns	ns	SPA/IP	DS	SC	ME	P	Parsonage and Hughes (2002)
*Littoraria philippiana*	3	BC	ns	ns	SPA/IP	DS	SC	ME	P	Parsonage and Hughes (2002)
*Nucella lapillus*	2–6	BC, BA	ns	ns	SPA/IP	DS	STS	F, ME	D	Etter (1988), Harris and Jones (1995)
*Nucella emarginata*	3	BC	MGT	ns	SPA	ns	ns	CE	D	Palmer (1984)
*Nucella ostrina*	ns	Color Brightness	ns	ns	ONT	PhP	ns	ME	D	de Bruyn and Gosselin (2014)
*Urosalpinx cinerea*	3	BC	MGT	ns	IP	ns	ns	CE	D	Cole (1975)
*Oliva oliva*	3	BC	ns	ns	IP	PhP	ns	F	D	Tursch et al. (1995)
*Asolene platae*	2	BC/BA	MGB	ns	IP	ns	ns	CE	D	Tiecher et al. (2017)
*Hastula cinerea*	3	BC	ns	ns	IP	DS	STS	F	D	Molina et al. (2013)
*Tegula xanthostigma*	2	BC	ns	ns	SPA	ns	ns	F	P	Yamazaki et al. (2019)
*Buccinum undatum*	29	BC/BA	ns	ns	SPAGR	ns	ns	F	D	Magnúsdóttir et al. (2018)
*Lottia asmi*, *L*. *digitalis*, *L. pelta*	3, ns, 6	BC/BA	ns	ns	IP, SPA	DS/PhP	SC	F, ME	P	Lindberg and Pearse (1990), Sorensen and Lindberg (1991)
*Batillaria attramentaria*	6	BC/BA	ns	ns	SPA	DS/GD	STS	F	D	Miura et al. (2007)
*Crepidula convexa*	3	BC	MGB	ns	IP	DS	SC	F	D	Hoagland (1977)
*Haliotis discus*	3	BC	MGT	ns	IP	DS/PhP	SC	CE	P	Liu et al. (2009)
*Concholepas concholepas*	Cryptic color	BC	ns	ns	IP	DS/PhP	SC	ME	D	Manríquez et al. (2009)

Note

ns, not specified.

^a^ BC, Background Color; BA, Bands, spots and tesselations.

^b^ MG, Monogenetic; MGB, Monogenetic Biallelic; MGT, Monogenetic Triallelic; DG, Digenetic; TGB, Trigenetic Biallelic.

^c^ IP, Intrapopulation; SPA, Spatial; SPAGR, Spatial Gradient; SNL, Seasonal; ONT, Ontogenetic change in color.

^d^ DS, Direct Selection; GD, Genetic Drift / Neutralism; NFDS, Negative Frequency‐Dependent Selection; BS, Balancing Selection; HS, Habitat Selection; PS, Pleiotropic Selection; SS, Sexual Selection; PhP, Phenotypic Plasticity.

^e^ SC, Selection for Crypsis; APS, Apostatic Selection; SHS, Selection against Haline Stress; STS, Selection against Thermal Stress; DM, Dissortative Mating/Negative Assortative Mating.

^f^ F, Frequentist; CE, Crossing Experiments; ME, Manipulative Experiments; CS, Cross‐Sectional design; MP, Model Predictions; MOC, Mother‐Offspring Correlation; ‐*Omics*, genomic, transcriptomic and/or proteomic techniques; RS, Reflectance Spectrometry.

^g^ D, Direct development; P, pelagic larvae.

## 5 METHODOLOGICAL OPTIONS

The methodology used to study a phenomenon may itself condition its understanding. For this reason, it is convenient to take into account the kinds of methodologies used when studying shell color polymorphism in order to detect some potential biases or limitations. Several methodologies exist, depending on the goals of the study (see Table 2). If our goal is the physical determination of color, or the precise characterization of color phenotypes, then these can either be scored visually by the researcher (e.g., against a color palette), analyzed based on digital images (e.g., RGB scale), or examined with reflectance spectrometry (RS; when the color is objectively estimated by a spectrometer through wave length reflectance). If we seek to analyze the molecular basis of shell color polymorphism, the pigments responsible for the different colors can be studied, for instance, through high‐performance liquid chromatography (HPLC) or mass spectrophotometry (Saenko & Schiltuizen, 2021). If our goal is to understand the hereditary basis of shell color polymorphism, then we can employ crossing experiments (CE; when using breeding experiments in the laboratory to infer Mendelian‐like proportions), “‐*Omics*” approaches (OA; based on genomic, transcriptomic or proteomic data), or knockout experiments. Although knockout experiments or other gene‐editing techniques have never been applied to the study of shell color polymorphism in marine gastropods, they have yielded interesting results when applied to other mollusk traits, such as biomineralization of the shell (Clark et al., 2020). In addition, and even though studies done so far strongly suggest that color is a genetically‐codified trait, epigenetic approaches may help explain those shell color traits most difficult to explain by means of genetic mechanisms or that are related to plastic mechanisms. These possibilities have been discussed in other invertebrate taxons (e.g., Hiyama et al., 2012). Finally, if our goal is to ascertain the evolutionary mechanisms at play in the maintenance of shell color polymorphism, various approaches can be followed: frequentist (F; when the emphasis is made on the distribution of color frequencies within or across populations), manipulative experiments (ME; any field or laboratory experiment based on the deliberate control of potential causal factors), model predictions (MP; those approaches that develop computer‐based explanatory models, especially when they are tested against empirical data) and cross‐sectional or fitness estimation designs (CS; when color frequencies are compared at different life‐cycle stages).

**TABLE 2:** Methods for the study of shell color polymorphism depending on different goals

Goals	Methodology
*Physical determination*	Visual scoring
	Digital imaging
	Reflectance spectrometry (RS)
*Molecular characterization*	High‐performance liquid chromatography (HPLC)
	Mass spectrophotometry
*Hereditary basis*	Crossing experiments (CE)
	Knockout experiments
	Omic approaches
	Epigenetic approaches
*Evolutionary mechanisms*	Frequentist studies (F)
	Manipulative experiments (ME)
	Model predictions (MP)
	Cross‐sectional designs (CS)

Each of these methods has its own strengths and limitations. For instance, ME are especially valuable to ascertain the causal role of the different evolutionary factors putatively involved in the maintenance of a polymorphism. However, ME are generally (though not always) carried out in the laboratory since there it is easier to control for intervening variables; but this raises several problems of its own. First, ME are only well‐suited for organisms that can be bred and kept in laboratory conditions, thus limiting the spectrum of organisms amenable for this kind of studies. Second, it poses the problem of generalization, given that laboratory settings are highly artificial and controlled and it is not always easy to extrapolate those results to natural environments (Ruxton & Colegrave, 2006). On the other hand, when we want to avoid these limitations by carrying out a ME in the field, we are confronted with a lot of practical setbacks related to the difficulty of controlling for intervening causal variables from the natural environment. On the contrary, frequentist (F) studies, given their observational and/or correlational nature, can be easily done in the field. However, their main limitation has to do with a lack of causal control, which makes drawing causal inferences more difficult. Model Predictions (MP) allow the testing of a hypothesis in a straightforward way. Nevertheless, they sometimes made dubious assumptions that limit their explanatory scope.

In sum, there is no perfect method for studying shell color polymorphism. Researchers should think carefully about the available methodological options and weight them against each other in order to determine which is the best methodology for a specific goal or biological question.

## 6 FUTURE OF THE FIELD

A considerable amount of knowledge has been gathered in the past decade regarding shell color polymorphism in marine gastropods (Table 1). However, many questions remain unanswered. In relation to the future of the field, and based on what we have previously exposed throughout this review, we propose new directions and goals to which new researchers could direct their efforts in order to take this field forward. These are centered around certain methodological issues and evolutionary mechanisms.

### 6.1 Methodological Issues

According to Table 1, most studies done so far have followed a frequentist (F) approach (25 out of 61) and several of these show the existence of a correlation between shell colors and their various backgrounds. Looking ahead, frequentist studies can be still very useful if, for example, frequentist geographical data are combined with detailed geographic‐scale environmental information, in order to detect correlations that could be interpreted in the light of potential causal relationships with environmental variables. Moreover, to date no study has yet analyzed the temporal consistency of geographic color morph frequencies in marine gastropods over decades, similarly to how Ramos‐Gonzalez and Davison (2021) have recently done in *Cepaea nemoralis* populations from the Pyrenees. Although a few studies exist that focus on temporal variation in shell color frequency (see Estévez et al., 2020, 2021; Johannesson & Butlin, 2017), this approach could be of interest not only to determine how evolutionary forces operate over long periods of time, but also because it may give new insights on the impact of color polymorphism on the vulnerability of a species (Bolton et al., 2015, 2016; Forsman, 2016; Takahashi & Noriyuki, 2019). In this sense, given that some studies suggest that shell color may contribute to body thermoregulation, it would be very interesting to ascertain if there has been any kind of selective process due to environmental variation over long time scales, as this is directly linked to climate change research (e.g., Leung et al., 2019; Minuti et al., 2021; Oliveira et al., 2020). Such a process has already been described in some species of insects (Brakefield & de Jong, 2011; de Jong & Brakefield, 1998).

The second kind of most conducted studies as derived from Table 1 are manipulative experiments (ME) (21 ME vs 25 F). Despite all their problems, ME have proven to be very useful, allowing researchers to infer causal relationships between shell color polymorphism and the different evolutionary mechanisms. So we argue researchers must keep doing MEs. In order to avoid the problem of generalization, efforts should be directed toward manipulative experiments in the field, or at least mesocosms experiments, as only they guarantee the ecological validity of results. Tethering and caging experiments would be the most obvious based on previous studies, although adaptation of experimental protocols from other taxons should not be ruled out (e.g., Endler, 1980).

Another important methodological consideration for future studies refers to shell color characterization by researchers. To date, most studies in marine gastropods have analyzed shell color using a visual scoring method (i.e., the observational criterion of experts guided by color templates; e.g., Pettitt, 1973), that is, a qualitative method. Although valuable as a first approximation, this method is subjected to several potential biases, such as low reliability. Therefore, we recommend the use of reflectance spectrometry (RS), as well as other potential quantitative approaches, to study shell color polymorphism. RS has only been used a few times in marine gastropods (Dauphin & Denis, 2000; McKillup & Mckillup, 2008; Rolán‐Alvarez et al., 2015b). In addition to render more robust and quantitative determinations of shell color, it can help to detect patterns of reflectance in the non‐visible spectrum, like the ultraviolet range.

Finally, this review has highlighted the lack of studies using ‐*Omics* approaches (OA) (genomic, transcriptomic, and proteomic) to unravel the genetic basis of color polymorphism in marine gastropods (2 studies of 61). Crossing experiments (CE) have shown that shell color in these species could have a rather simple genetic basis (e.g., one to a few loci). This suggests that although the genotyping of families bred in the laboratory or genome‐wide association studies (GWAS) represent very useful methods to determine the genetic basis of color, it still represents a daunting task, especially when a reference genome is not available in the species, and thousands of genetic markers (e.g., SNPs) are ideally needed (Koch et al., 2021). One of the reasons for the lack of such studies could be the relatively large size of the genome of these animals, with a lot of them being greater than 1.0 Gb and some having even larger genomes (e.g., dogwhelks 2.8 Gb; www.genomesize.com).

Another important reason is that until very recently whole‐genome sequencing projects have been associated with model species or those with an economic interest, although nowadays these kinds of projects have also been carried out in species with an ecological or evolutionary interest (e.g., *Littorina saxatilis*, Westram et al., 2018). It is predicted that these ‐*Omics* studies will considerably increase in the next years with the availability of high‐quality genome assemblies for a growing number of species, including marine gastropods, lower sequencing prices, and new knowledge about the relationship between shell color polymorphism and the different evolutionary mechanisms in marine gastropods. Certainly, a combination of ‐*Omics* studies with CE would be ideal to determine in the future the precise genetic basis of shell color and its polymorphism.

In recent years, GWAS type analyses have been widely used to study color polymorphism as a start point to depict the genomic regions associated with a specific color. Methodologically this has been done by WGS (Whole Genome Sequencing) of DNA pools of each color (e.g., Lopes et al., 2016, in canary birds; Gautier et al., 2018, in ladybirds; Andrade et al., 2019, in lizards), or by GBS (genotyping by sequencing) of individual samples and then combining the genotypes of each specific color (e.g., Nosil et al., 2020, in stick insects). The use of families from CE in the laboratory (e.g., Koch et al., 2021) is more powerful than GWAS, but it is also a lot more time consuming because of the breeding stage in the laboratory, which is highly dependent on the organism (marine gastropods are not ideal in that sense). In any case, what all the previous studies have in common is the use of a reference genome to map the short sequences obtained by NGS (Next Generation Sequencing), in some cases being an already existing genome in the databases, and in others a newly sequenced genome specifically for that study. If we focus on our phylum of interest, that of mollusks, transcriptomic studies mostly in bivalves of economic interest have been relatively common (e.g., Ding et al., 2015; Hu et al., 2019; Xu et al., 2019). These studies focus on the differential expression of genes across the whole transcriptome between different colors, assuming that a part of those transcripts would be candidates for being involved in the inheritance of color. Following these analyses, the functional annotation of the transcripts is necessary and the quality of this annotation is a key step in order to study the different functions of the candidates. Some studies (e.g., Xu et al., 2019) also include proteomic analyses (e.g., mass spectrometry‐based proteomics) to support the transcriptomic results. It has been observed in these mollusk studies that the number of differentially expressed genes is large and that finding good gene candidates is difficult. Finally, although genomic studies might be more effective at the time of finding a candidate genomic region than candidate genes through transcriptomics, these genomic regions are usually rather large and study of gene expression of genes present in that region represents a common follow up of the GWAS analysis (Andrade et al., 2019; Gautier et al., 2018; Lopes et al., 2016). Therefore, even with the availability of NGS technologies, targeting a gene or a few causal genes for color polymorphism still represents a great challenge.

### 6.2 Evolutionary mechanisms

As we have already seen, there are plenty of studies that have analyzed the putative evolutionary mechanisms that may be maintaining shell color polymorphism in marine gastropods; however, several facts stand out. First, not all evolutionary mechanisms have been studied equally, direct selection has received much more attention than other selective and non‐selective mechanisms (30 out of 61 studies). A conclusion that can be drawn from Table 1 is that the most frequent mechanism used to explain color polymorphism has been natural selection in its different guises (crypsis, apostatic, mate choice, etc.). This may be justified due to the heuristic potential of adaptive hypotheses (Mayr, 1983); however, we believe that each hypothesis deserves its fair chance. A very useful approach to solve this would be to explicitly employ the equilibrium between genetic drift and migration as null hypothesis of selective mechanisms when conducting evolutionary studies.

Second, the pleiotropic and genetic linkage hypotheses have a great but still unexplored explanatory potential, since suggesting evidence reviewed in previous sections points to the possibility that, at least in some marine gastropod species, shell color may not be the real target of selection. However, most of this research ultimately requires unraveling information about the genetic basis of shell color, and would demand more efforts in generating genomic data for those species that might show this kind of patterns. This explanatory recommendation is closely linked, therefore, to the abovementioned need of future studies to focus more on ‐*Omics* techniques.

Thirdly, in the past decade it has been suggested that the evolution and ecological performance of genetically polymorphic species (ecologically associated) may be greater than those of plastic (environmentally caused) polymorphic species, or than those species in which the association between the polymorphism and the environment is random (Forsman et al., 2008; but see Bolton et al., 2015, 2016). These studies suggest that more geographically variable species show broader niches, reduced intra‐specific competition, decreased vulnerability to environmental changes (or genetic drift), and decreased extinction risk. Other authors have claimed that marine gastropods may have a special capability to resist at least some effects of climate change, such as temperature increases (Leung et al., 2019). We suggest that shell color polymorphism in marine gastropods may also be of great value for checking these hypotheses. For instance, as suggested in the previous section, if certain color morphs resist thermal stress better (as studies suggest; see section 4.2.1.), and if temperatures rise in certain populations due to climate change, then an increase in the frequency of such morphs in those populations can be expected.

Finally, we are currently witnessing a renewal of evolutionary theory, with promising new ideas and mechanisms, such as genetic assimilation, epigenetic inheritance, evo‐devo, or niche construction, that are receiving increasing attention (Laland et al., 2015; Pigliucci & Müller, 2010). The study of shell color polymorphism in marine gastropods cannot leave behind these new trends in evolutionary biology. For example, phenotypic plasticity in gastropods is an established fact (Bourdeau et al., 2015), and here we have seen some putative cases of plastic shell color polymorphism in marine gastropods. However, its role as an evolutionary mechanism is not clear, because the possibility of genetic assimilation of plastic traits has not been yet evaluated in this group of organisms. Putative cases of genetic assimilation in other groups of animals (e.g., Aubret & Shine, 2009; Badyaev et al., 2017) may give us clues as how to test such hypothesis for shell color polymorphism in marine gastropods.

As a final commentary, we must say that, after reading this review, some may worry that, to date, “the” evolutionary mechanism behind shell color polymorphism in marine gastropods has not yet been resolved in many cases. However, as a philosopher of science John Beatty (1997) has argued, disputes in biology often revolve around the question of how many cases in a particular domain of phenomena does one theory or mechanism explains. Or, in other words, what is its *relative significance*. Applied to this case, the really pressing question is not what is “the” actual evolutionary mechanism responsible for shell color, but how many instances of shell color polymorphism does each evolutionary mechanism explain. Evolution has many ways of arriving at a particular scenario, so we should not expect to find a single answer to the causes of such a huge diversity of shell color polymorphisms that we observe in nature. Instead, we should adopt a more pluralist outlook and be open about the potential contribution of different evolutionary mechanisms to the explanation of shell color polymorphism.

## 7 CONCLUSION

Shell color polymorphism in marine gastropods is a pervasive phenomenon. Throughout the last decade, great advances have been made in the understanding of the different aspects that comprise it. In this review, we have summarized such knowledge. In particular, we have focused our attention on the evolutionary forces that could be contributing to its maintenance in natural populations. We have also derived from such knowledge a series of recommendations regarding the most promising methodological options, as well as the more fertile evolutionary explanations, given current limitations. Although a quick glance at the explanatory mechanisms suggests that natural selection may have a prominent role in explaining shell color polymorphism in several marine gastropods, some gaps in our current knowledge of such phenomenon exist. Unraveling the evolutionary mechanisms responsible for the existence and maintenance of intraspecific variation—including color polymorphism– is a major topic of study in evolutionary biology, as they are the key to biodiversity. Shell color polymorphism in marine gastropods represents a well‐suited model system to study those mechanisms, and the new ‐*Omics* techniques constitute a promising approach to unraveling those evolutionary mechanisms at the molecular level. For this reason, we urge evolutionary biologists and malacologists alike to focus their efforts on the study of the various aspects of shell color polymorphism in this diverse and beautiful group of organisms.

## CONFLICT OF INTEREST

The authors declare they have no conflict of interest.

## Data Availability

Data sharing not applicable—no new data generated.
